# The genome of *Naegleria lovaniensis*, the basis for a comparative approach to unravel pathogenicity factors of the human pathogenic amoeba *N. fowleri*

**DOI:** 10.1186/s12864-018-4994-1

**Published:** 2018-09-05

**Authors:** Nicole Liechti, Nadia Schürch, Rémy Bruggmann, Matthias Wittwer

**Affiliations:** 1 Interfaculty Bioinformatics Unit, University of Bern, Bern, Switzerland; 2 Biology Division, Spiez Laboratory, Federal Office for Civil Protection, Austrasse, Spiez, Switzerland; 3 Graduate School for Cellular and Biomedical Sciences, University of Bern, Bern, Switzerland

**Keywords:** *Naegleria lovaniensis*, *Naegleria fowleri*, PacBio sequencing, Genome de novo assembly, Primary amoebic meningoencephalitis, Comparative genomics

## Abstract

**Background:**

Members of the genus *Naegleria* are free-living eukaryotes with the capability to transform from the amoeboid form into resting cysts or moving flagellates in response to environmental conditions. More than 40 species have been characterized, but only *Naegleria fowleri* (*N. fowleri*) is known as a human pathogen causing primary amoebic meningoencephalitis (PAM), a fast progressing and mostly fatal disease of the central nervous system. Several studies report an involvement of phospholipases and other molecular factors, but the mechanisms involved in pathogenesis are still poorly understood. To gain a better understanding of the relationships within the genus of *Naegleria* and to investigate pathogenicity factors of *N. fowleri*, we characterized the genome of its closest non-pathogenic relative *N. lovaniensis*.

**Results:**

To gain insights into the taxonomy of *Naegleria*, we sequenced the genome of *N. lovaniensis* using long read sequencing technology. The assembly of the data resulted in a 30 Mb genome including the circular mitochondrial sequence. Unravelling the phylogenetic relationship using OrthoMCL protein clustering and maximum likelihood methods confirms the close relationship of *N. lovaniensis* and *N. fowleri.* To achieve an overview of the diversity of *Naegleria* proteins and to assess characteristics of the human pathogen *N. fowleri*, OrthoMCL protein clustering including data of *N. fowleri*, *N. lovaniensis* and *N. gruberi* was performed. GO enrichment analysis shows an association of *N. fowleri* specific proteins to the GO terms “Membrane” and “Protein Secretion.”

**Conclusion:**

In this study, we characterize the hitherto unknown genome of *N. lovaniensis.* With the description of the 30 Mb genome, a further piece is added to reveal the complex taxonomic relationship of *Naegleria*. Further, the whole genome sequencing data confirms the hypothesis of the close relationship between *N. fowleri* and *N. lovaniensis*. Therefore, the genome of *N. lovaniensis* provides the basis for further comparative approaches on the molecular and genomic level to unravel pathogenicity factors of its closest human pathogenic relative *N. fowleri* and possible treatment options for the rare but mostly fatal primary meningoencephalitis.

**Electronic supplementary material:**

The online version of this article (10.1186/s12864-018-4994-1) contains supplementary material, which is available to authorized users.

## Background

*Naegleria* spp. are free-living amoebas of the class Heterolobosea and are found in soil and fresh water sources all over the world. As amoeboflagellates, they are able to transform under changing environmental conditions from the amoeboid form into fast moving flagellates or resting cysts [1]. Based on the analysis of internal transcribed spacer sequences located on the ribosomal DNA, over 40 species have been characterized [2]. So far only one species, *N. fowleri* is known as a human pathogen causing primary amoebic meningoencephalitis (PAM), a fast progressing and mostly fatal disease of the central nervous system [3]. Infections with *N. fowleri* occur when contaminated water, for example during swimming or ritual nose cleansing, enters the nose and the amoeba find its way along the olfactory nerves to the brain by crossing the cribriform plate [4, 5]. The progression of the disease is rapid, and most patients die within 14 days. In many cases, the diagnosis is made post mortem [6]. While the worldwide prevalence is low, in several countries (e.g. Pakistan) the number of reported cases increased in the last few years [7]. Several studies report an involvement of phospholipases, proteases and other molecular factors, but the mechanisms involved in the pathogenesis are poorly understood. Although the genome of *N. fowleri* was published in 2014 in connection with a comparative proteomics study of high versus low pathogenic amoebae [8], there is still little known about *Naegleria* on the genomic and transcriptomic level. One of the best characterized species is the non-pathogenic and non-thermotolerant *N. gruberi*. It serves as a model for basal body and flagellar assembly processes and its genome was published in 2008 [9]. Since *N. gruberi* is well studied as a model organism, several groups assessed pathogenicity factors using a comparative approach, for example by comparing protease activities [10] or membrane glycoconjugates [11] between the human pathogenic *N. fowleri* and the non-pathogenic *N. gruberi*. The suitability of *N. gruberi* as a close non-pathogenic model is questioned by the work of Herman et al. [12] analysing the mitochondrial sequences and a 60 kb nuclear segment of *N. fowleri* and *N. gruberi*. The comparison of these genomic features showed less synteny than expected [12]. Therefore, accessing virulence factors by an interspecies comparison of *N. fowleri* and *N. gruberi* on a molecular and genomic level may result in misleading findings. Moreover, phylogenetic analysis of *Naegleria* based on ribosomal DNA and internal transcribed spacer sequences gives evidence, that *N. lovaniensis* is closer related to *N. fowleri*. Due to the high sequence similarity, it is even probable that *N. fowleri* and *N. lovaniensis* evolved from a common ancestor [2]. Summarized, there is still a lack of knowledge regarding phylogenetic relationships as well as the diversity on a genomic level. However, to unravel mechanisms involved in pathogenesis, knowledge of the genomic structure and taxonomy of the Genus *Naegleria* are crucial. In the last few years, Next Generation Sequencing (NGS) Technologies evolved rapidly. Besides short read sequencing, new methods with substantially increased read length such as Single Molecule Real Time sequencing have gained more importance. With the ability of spanning repetitive sequences, long reads provide new possibilities in the field of de novo genome assembly. New bioinformatics tools for genome de novo assembly have also been established [13, 14]. Long read assemblers implement approaches that consider the heterozygosity of genomes and comprise algorithms for the correction of sequencing errors. They are powerful tools for de novo assembly of eukaryotic genomes. To gain insights into the diversity of *Naegleria* focusing on the pathogenicity of *N. fowleri*, we characterize the genome of the close non-pathogenic relative *N. lovaniensis*. With the assembly of the *N. lovaniensis* genome, a further reference is added to the class of Heterolobosea and provides insights into the complex phylogenetic structure of the genus of *Naegleria*. The sequencing data support the close relationship of *N. fowleri* and *N. lovaniensis*. Furthermore, the characterization of *N. lovaniensis* provides the basis for further comparative approaches on the molecular and genomic level to unravel pathogenicity factors of *N. fowleri*. Analysis of *N. fowleri* specific protein clusters identified members of Rab and Rho small family GTPases, which are involved in vesicular trafficking and cytoskeletal reorganization. Furthermore, our data highlight the importance of actin adhesion structures and secretory processes during *N. fowleri* pathogenesis.

## Methods

### Cultivation of *N. lovaniensis*

*N. lovaniensis* trophozoites (ATCC #30569) were cultivated at 36 °C in different media types according to Burri et al. [15] using Nunclon™ Δ Surface cell culture flasks (Thermo Fisher Scientific, Allschwil, Switzerland). Nelson’s Medium contains 0.1% (*w*/*v*) Liver Hydrolysate (Sigma, Buchs, Switzerland), 0.1% (w/v) D-(+)-glucose (Sigma), and 10% (*v*/v) fetal calf serum in Page’s amoeba saline (2 mM NaCl, 16 μM MgSO_4_, 27.2 μM CaCl_2_, 1 mM Na_2_HPO_4_, 1 mM KH_2_PO_4_). PYNFH medium consists of 1% (*w*/*v*) Bacto peptone (BD Biosciences), 1% (w/v) yeast extract (BD Biosciences), 0.1% (w/v) yeast ribonucleic acid (Sigma), 15 mg folic acid (Sigma) and 1 mg haemin (Sigma) supplemented with 10% (*v*/v) fetal calf serum in 133 mM KH_2_PO_4_, 176.1 mM Na_2_HPO_4_. As a third media type, PYNFH medium was supplemented with 0.1% (w/v) Liver Hydrolysate (Sigma, Buchs, Switzerland).

### Sequencing of genomic DNA

For DNA isolation, *N. lovaniensis* was cultivated in Nelson’s Medium, trophozoites were detached from culture flask using a cell scraper and harvested by centrifugation followed by three washing steps using Dulbecco’s phosphate-buffered saline (PBS, Sigma). DNA was extracted using DNeasy Blood and Tissue Kit (Qiagen, Basel, Switzerland) according to the manufacturer’s protocol. Any remaining RNA was digested using RNase A (Qiagen). The DNA was eluted in 100 μl EB Buffer (10 mM Tris-Cl, pH 8.5, Qiagen) preheated to 70 °C. Total DNA was quantified using the Qubit 2.0 Fluorometer with the Qubit dsDNA HS Assay Kit (Thermo Fisher Scientific). Further, the quality was analysed using the Agilent 2100 Bioanalyzer system with the Agilent DNA 12000 Kit (Agilent Technologies, Basel, Switzerland). High molecular-weight DNA (18 μg) was sent to the Functional Genomic Centre (Zurich, Switzerland) for library preparation and PacBio Sequencing of 10 SMRT cells using P6C4 chemistry on RSII platform.

### Sequencing of RNA

For total RNA isolation, *N. lovaniensis* was cultivated in the media Nelson, PYNFH and PYNFH supplemented with Liver Hydrolysate. Trophozoites were harvested in the late log phase by centrifugation and washed three times using PBS. For cell disruption, trophozoites were resuspended in 750 μl QIAzol lysis reagent (Qiagen) and homogenized in the TissueLyser (Qiagen) for 3 min at 25 Hz. After incubation at room temperature for 5 min, 150 μl chloroform (Sigma) was added to the sample followed by centrifugation for 15 min at 12,000 g at 4 °C. RNA from the upper aqueous phase was extracted using the EZ1 RNA Universal Tissue Kit (Qiagen) and the EZ1 BioRobot (Qiagen) according to the manufacturer’s protocol. The purified RNA was quantified using the Qubit2.0 Fluorometer with the Qubit RNA HS Assay Kit (Thermo Fisher Scientific) and the total RNA integrity was examined using the Agilent 2100 Bioanalyzer system (Agilent Technologies). Total RNA (2 μg) was sent to the NGS Platform at the University of Bern (Bern, Switzerland) for Illumina HiSeq 3000 150 bp paired-end sequencing.

### De novo genome assembly

#### De novo assembly using FALCON

A de novo assembly of the *N. lovaniensis* genome was performed using FALCON (v.0.7.0) [14]. FALCON is a string graph assembler that uses in a first step DALIGNER [16] to compare overlapping reads and to generate high accurate consensus sequences of the longest reads with a predefined length cut off. In a second step, a string graph is constructed using the overlaps of the consensus sequences. The string graph is then reduced to primary and associative contigs representing heterozygous sequences [14]. To assemble the *N. lovaniensis* genome, the length cut off used for initial mapping was auto-calculated by setting the genome size to 30 Mb, while the length cut off for the pre-assembly was set to 5000. For the overlap filtering max_diff was set to 100, min_cov was set to 4, and max_cov was set to 200 to retrieve repetitive regions, the rDNA plasmid sequence and the mitochondrial sequence. To improve the genomic consensus, the assembly was polished using Quiver [17]. Additionally, the mitochondrial sequence was circularized manually.

#### Quality assessment of the genome assembly

To assess the completeness of the de novo assembled genome, BUSCO v2 [18] was used to search for a set of 303 conserved eukaryotic Benchmarking Universal Single-Copy Orthologs [19]. To validate the output of BUSCO and for comparison between *Naegleria* species, the tool was applied to the previous published genomes of *N. fowleri* and *N. gruberi*.

#### Assembly and annotation of the rDNA plasmid

Due du its high repeat content, the rDNA plasmid cloud not be fully assembled in the initial FALCON assembly. To improve the assembly, raw reads were mapped to the 12 kb rDNA fragment assembled by FALCON using minimap2 [20]. Mapped reads were assembled using Canu 1.7 [13] followed by Quiver polishing and manual circularization. Non-coding RNAs and rRNA sequences were annotated using INFERNAL 1.0.2 [21] and BLAST to the *N. gruberi* annotated rDNA plasmid (Accession: MG699123.1) as well as to a public available *N. lovaniensis* ribosomal sequence (Accession:MH304644).

### Genome annotation

#### Repetitive elements

Repetitive elements were predicted using a de novo approach by applying RepeatModeler [22], which includes RECON [23] and RepeatScout [24] to construct a *Naegleria*-specific repeat library. To further classify repetitive sequences, Hmmer3.1b1 [25] was used to search for protein domains and known LTR domains. Sequences with similarities to known proteins were discarded from the repeat library. Furthermore, repetitive sequences were submitted to TEclass [26] to categorize the sequences into DNA transposons, LTRs, and non-LTRs. In a second step, RepeatMasker [27] was applied to mask repetitive sequences using the de novo constructed library.

### Gene annotation

**Fig. 1:**
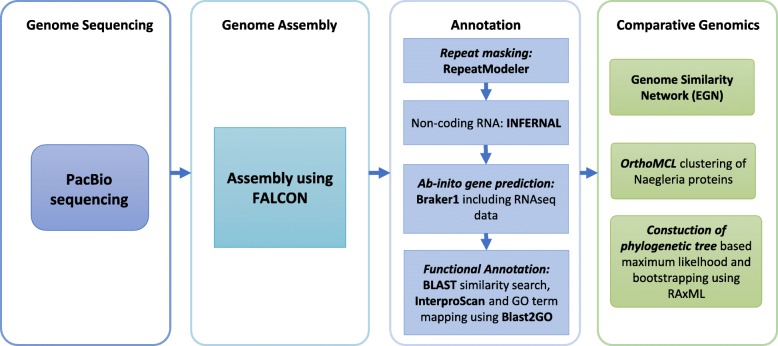
Workflow Genome de novo Assembly. To achieve a complete as possible assembly of the *N. lovaniensis* genome, PacBio data were assembled using FALCON quality and completeness assessment using BUSCO. Repetitive sequences were identified and masked by RepeatModeler. In a further step, non-coding RNAs were identified using INFERNAL and proteins were predicted by an ab-initio method applying BRAKER1 including RNAseq data. Functional annotation was performed using Blast2GO pipeline. To gain an overview of *Naegleria* species, a genome similarity network as well as a phylogenetic tree was constructed. Additionally, *Naegleria* proteins were clustered using OrthoMCL

Protein coding genes on the nuclear genome were predicted using an ab-initio approach taking into account RNAseq data by applying BRAKER1 [28]. BRAKER1 uses GeneMark-ET [29] to generate ab-initio gene models by unsupervised learning including RNAseq data. In a second step the gene models are used to train AUGUSTUS [30]. AUGUSTUS uses unassembled RNAseq data for the final gene prediction. Non-coding RNAs were annotated by searching the Rfam 12.1 database [31] for sequence similarities using INFERNAL 1.0.2. Functional annotation of predicted proteins was done by BLAST similarity search of the predicted proteins against SwissProt and UniRef90 [32] with an e-value of 1e-05. The top five BLAST hits were loaded into CLC Genomics Workbench 9.5.2 (Qiagen) and GO terms were retrieved using Blast2GO [33] plug-in with default annotation parameters. To further improve the annotations, InterproScan was used within CLC to identify known protein domains. BLAST results of both databases and results of the InterproScan analysis were combined to gain a complete functional annotation of all predicted genes. Additionally, PFAM domain search was performed separately against PFAM-A 29.0 [34] using HMMER v3.1b2. Figure 1 depicts a summary of the de novo genome assembly and annotation workflow.

### Genome similarity

To gain an overview of the taxonomic relationship of *Naegleria*, a BLAST-based genome similarity network was constructed using Evolutionary Gene and Genome Networks software (EGN) [35]. The similarity network contains predicted proteins of *N. lovaniensis* and TransDecoder predicted Open Reading Frames (ORFs) of *N. fowleri* [8]. Additionally, the proteomes of *N. gruberi* (UP000006671), two human pathogenic amoebas, *E. histolytica* (UP000001926) and *A. castelanii* (UP000011083), as well as *T. brucei* (UP000008524) and *T. cruzi* (UP000017861), both belonging to the related group of Euglenozoans, were retrieved from UniProt. To identify shared protein families, an all-versus-all BLAST search was performed within EGN. Similar sequences were clustered by applying an e-value of 1e-5 and a hit identity of 50%. The network was visualized using Cytoscape 3.0.2 [36].

### Clustering of orthologous genes

To identify orthologs between different *Naegleria* species and to estimate the degree of relatedness, proteins of *N. lovaniensis, N. gruberi* (UP000006671) and *N. fowleri* were clustered using OrthoMCL [37]. For BLAST similarity search an e-value of 1e-5 and a minimal identity of 50% were applied.

### Phylogenetic analysis

To construct a phylogenetic tree, random 100 single-copy orthologs of the OrthoMCL clustering were aligned using MUSCLE [38]. Alignments were trimmed using trimAl [39] and concatenated to a supermatrix using FASconCAT [40]. Prottest3 [41] was used to select the best model for amino acid replacements, the phylogenetic tree was constructed based on maximum likelihood and bootstrapping using RAxML 8.1.2 [42] including 1000 bootstraps. The resulting tree was visualized with FigTree [43].

### Gene set enrichment analysis

To identify the function of *N. fowleri* and *N. lovaniensis* specific proteins, GO enrichment analysis was performed using the Cytoscape plug-in BiNGO [44]. As reference set for GO enrichment analysis, all functionally annotated proteins of the respective species were considered. Over-represented GO terms (Bonferroni-corrected *p*-value < 0.05) were examined using classical hypergeometric test.

## Results

### Genome assembly

To achieve a nearly complete genome assembly, total DNA of *N. lovaniensis* (ATCC #30569) was isolated and sequenced using the PacBio RSII sequencing platform. Sequencing of 10 SMRT cells resulted in 1,529,980 reads with a mean read length of 6893 bp after subread filtering. To reconstruct the genome, the data were assembled using FALCON, a diploid-aware string-graph assembler. To improve the consensus sequence and to correct insertions, deletions and substitution errors, raw reads were realigned to the assembly and the sequence was polished using Quiver. The final haploid assembly consists of 111 contigs with an N50 of 657,933 bp and has a total size of 30.8 Mb (30,838,059 bp). The genome of *N. lovaniensis* has a similar size as well as a similar GC content (37%) compared to the recently published *N. fowleri* genome. In contrast, the *N. gruberi* genome is slightly larger (40 Mb) and has a GC content of 35% [9] (Table 1). The completeness of the assembly was evaluated by searching for 303 eukaryotic Benchmarking Universal Single-Copy Orthologs (BUSCOs) [18]. BUSCOs evolved as single copy orthologs in a broad range of organisms; the eukaryotic set was selected based on OrthoDB v9.1 [45] data of 90 different species. Since the duplication or deletion of such evolutionary conserved single-copy genes are rare events, analysis of BUSCOs gives an overview of the completeness of de novo assembled genomes [18]. Analysing the *N. lovaniensis* genome, 265 (87.4%) BUSCOs were found in the final FALCON assembly, of which 6 are fragmented and 18 are duplicated. To benchmark the completeness of the assembly, the number of found BUSCOs was compared to those found in *N. fowleri* and *N. gruberi*. The comparison shows similar numbers of BUSCOs in the *N. fowleri* genome (total: 269, 88.8%) while less BUSCOs could be identified in the *N. gruberi* assembly (total: 257, 84.8%) (Table 2).

**Table 1:** Comparison of sequenced *Naegleria* genomes

	*N. lovaniensis*	*N. fowleri*	*N. gruberi*
Genome Size (Mb)	30.8	29.6	40.9
GC content (%)	37	37	35
Repeat content (%)	3.5	2.5	5.1
Number of contigs (Scaffolds)	111	1729 (574)	1977 (784)
N50 of contigs (bp)	657,933	38,128	159,679
Number of predicted genes	15,195	17,252 (based on RNAseq data)	15,711

**Table 2:** Comparison of BUSCO analysis of *Naegleria* genomes

	*N. lovaniensis*	*N. fowleri*	*N. gruberi*
Total Number of BUSCOs	265	269	257
Complete Single-Copy	247	265	253
Complete Duplicated	18	4	4
Fragmented	6	6	7
Missing	32	32	39

### Genome structure

The *N. lovaniensis* genome is gene dense, 77% of the genome is defined as coding sequences. Further, 47% of the predicted genes have at least one intron with a median length of 80 bp. Besides its nuclear DNA, the genome of *Naegleria* includes a circular mitochondrial sequence and an extrachromosomal plasmid encoding ribosomal RNAs [46]. The long-read assembly allowed the complete reconstruction of the mitochondrial genome sequence. With a size of 48,553 bp the mitochondrial genome of *N. lovaniensis* is shorter compared to the ones of *N. fowleri* (49,519 bp) and *N. gruberi* (49,842 bp). Furthermore, the extrachromosomal rDNA plasmid was assembled using Canu 1.7. With 15,131 kb the assembly length corresponds to the estimated size of the plasmid [47].

### Genome annotation

**Fig. 2:**
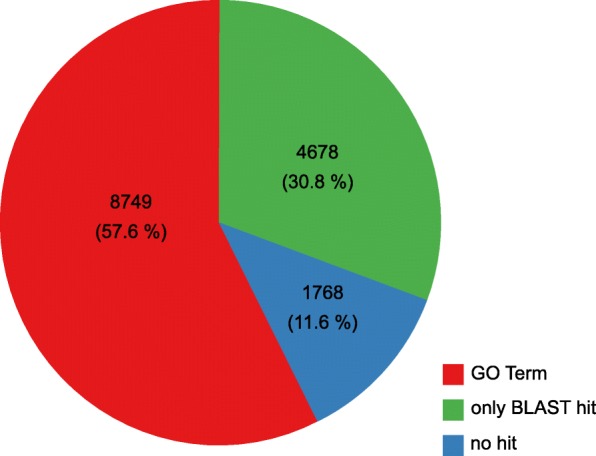
Overview of gene annotation of the *N. lovaniensis* genome**.** Of total 15,195 ab initio predicted proteins, 8749 (57.6%) proteins mapped to at least one GO term using Blast2GO; 4678 (30.8%) only have a BLAST hit, while 1768 (11.6%) show no similarity to known proteins or domains

Gene prediction in eukaryotes is still a challenge, especially in species with little information. *Naegleria* spp. are distantly related to other annotated species and lacking a well-established reference, de novo repeat prediction was performed using RepeatModeler to build a *Naegleria*-specific library. Approximately 3.4% of the total genome is classified as repetitive sequences. In total 1.25% is categorized as DNA transposons while 0.68% is classified as LTRs (Table 3). To accurately predict genes of the *N. lovaniensis* nuclear genome, an ab-initio method incorporating RNAseq data was applied. Using BRAKER1, a combination of GeneMark-ES and AUGUSTUS, 15,195 genes could be identified on the nuclear genome. In total, 13,005 (85.6%) of the predicted proteins contain at least one Pfam protein domain. The total number of predicted proteins is slightly lower compared to *N. gruberi* (15,727), while a higher number of proteins is predicted based on RNAseq data for *N. fowleri* (17,252) [8]. By applying INFERNAL 1.0.2 for non-coding RNA prediction, among others spliceosomal RNAs, U1-U6 as well as tRNA coding regions were identified. The functional annotation of the predicted proteins was performed by BLAST similarity search against SwissProt and UniRef90 by applying an e-value of 1e-05. GO terms were retrieved from BLAST results using Blast2GO CLC plug-in. The annotations were further improved using InterproScan to identify protein families. In total, 8749 (57.6%) proteins mapped to at least one GO term, 4678 (30.8%) only have a BLAST hit, while 1768 (11.6%) show no similarity to known proteins or domains (Fig. 2).

**Table 3:** Repetitive Sequences of the total genome

Class		Count	bp Masked	%masked
DNA		379	209,650	0.68%
	CMC-EnSpm	38	69,762	0.23%
	PiggyBac	29	23,595	0.08%
LINE		190	78,458	0.25%
LTR		208	115,388	0.37%
	DIRS	89	99,113	0.32%
	Gypsy	22	52,359	0.17%
Unspecified		57	20,784	0.07%
	total interspersed	1012	669,109	2.17%
Low_complexity		766	37,304	0.12%
Simple_repeat		7568	327,882	1.06%
Total		9346	1,034,295	3.35%

### Genome similarity

**Fig. 3:**
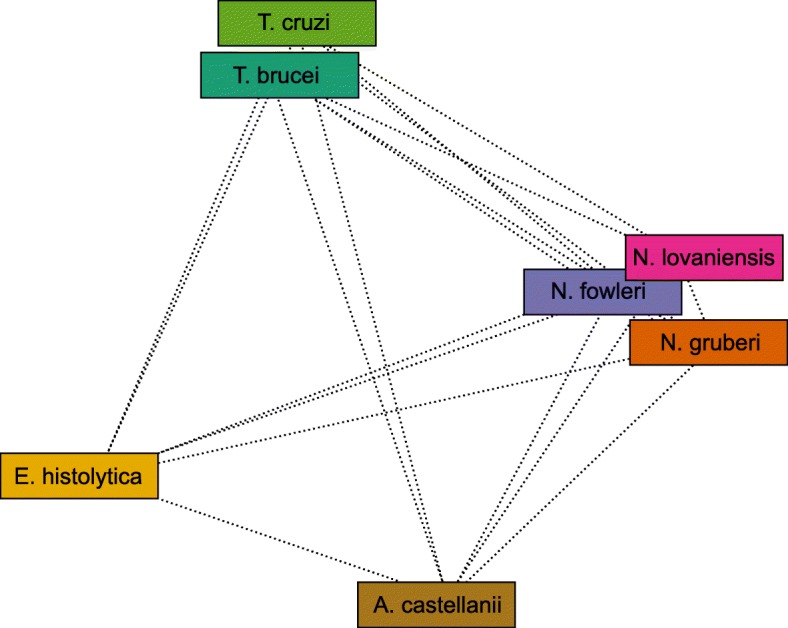
Genome Similarity Network of unicellular eukaryotic species**.** To gain insights of the phylogenetic relationship of *Naegleria*, a BLAST based Genome Network was constructed including *N. lovaniensis* and *N. fowleri* predicted protein as well as Uniprot proteomes of *T. brucei, T. cruzi, A. castelanii,* and *E. histolytica*. Nodes in the graph represent the organisms and the edges, which are inversely proportional to the number of shared gene families, are the measurement of similarity between nodes. Additional file 2 shows the number of shared gene families between species

**Fig. 4:**
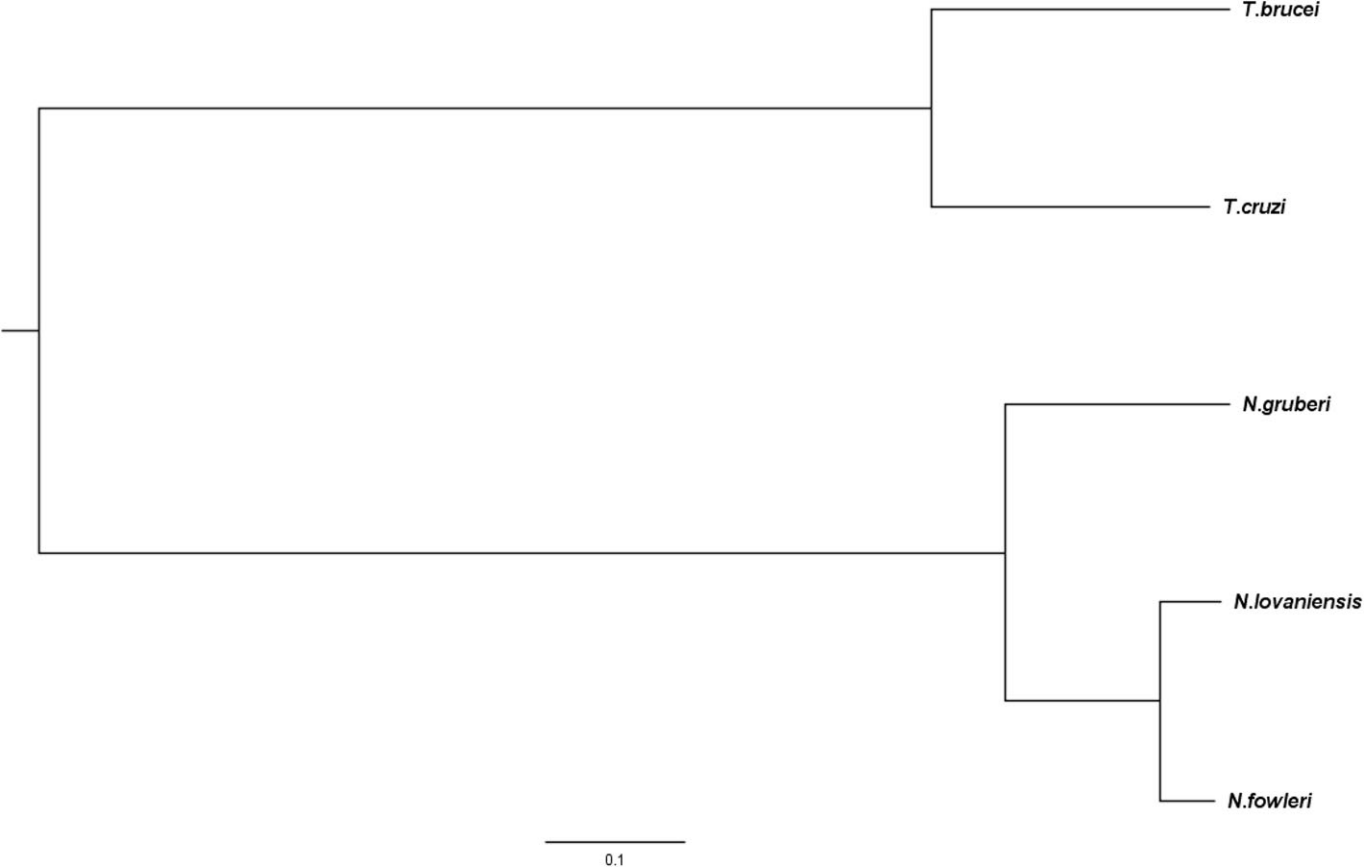
Maximum likelihood tree of *Naegleria* species. Based on maximum likelihood and bootstrapping using RAxML a species tree was constructed to achieve a comprehensive overview of the relatedness of the species within the genus of *Naegleria*. Phylogenetic distances were estimated based on 100 single copy orthologs defined by OrthoMCL clustering. Beside *Naegleria* species, the more distantly related protists *T. brucei* and *T. cruzi* were chosen as taxonomic outgroups

Unravelling the phylogenetic relationship of *Naegleria* is mandatory for further comparative studies on a genomic and molecular level, especially regarding the pathogenicity of *N. fowleri*. Therefore, we constructed a BLAST similarity-based genome network using Evolutionary Gene and Genome Networks software (EGN). Additional to the *Naegleria* species, two *Trypanosoma* species (*T. cruzi* and *T. brucei*), which belong to the related class Euglenozoa, and two human pathogenic amoebas (*A. castellanii* and *E. histolytica*) were included in the analysis. Within the network, nodes represent the organism and the length of the edges are proportionally inverse to the number of shared gene families. Regarding the cluster of *Naegleria*, *N. fowleri* shares 9547 genes with *N. lovaniensis*, while only 5831 genes are shared with *N. gruberi*. Furthermore, the network suggests a higher similarity between the cluster of *Naegleria* species and *A. castellanii* (527, *N. fowleri: A. castelanii*), a human pathogenic amoeba causing Granulomatous Amoebic Encephalitis, than to the trypanosomes of the related class Euglenozoa (233, *N. fowleri: T. brucei*) (Fig. 3, Additional file 1). To gain a comprehensive overview of the relatedness of *Naegleria* species, a phylogenetic tree based on maximum likelihood and bootstrapping using RAxML was constructed. Phylogenetic distances were estimated based on 100 single copy orthologs defined by OrthoMCL clustering of *Naegleria* species and the more distantly related protists *T. brucei* and *T. cruzi* as outgroups. Visualization of the tree provides a more detailed overview of the phylogenetic relationship. In concordance to the genome similarity network, the result shows a close relationship between *N. lovaniensis* and the pathogenic *N. fowleri*, while *N. gruberi* is more distantly related (Fig. 4).

### Clustering of gene families of *Naegleria* species

**Fig. 5:**
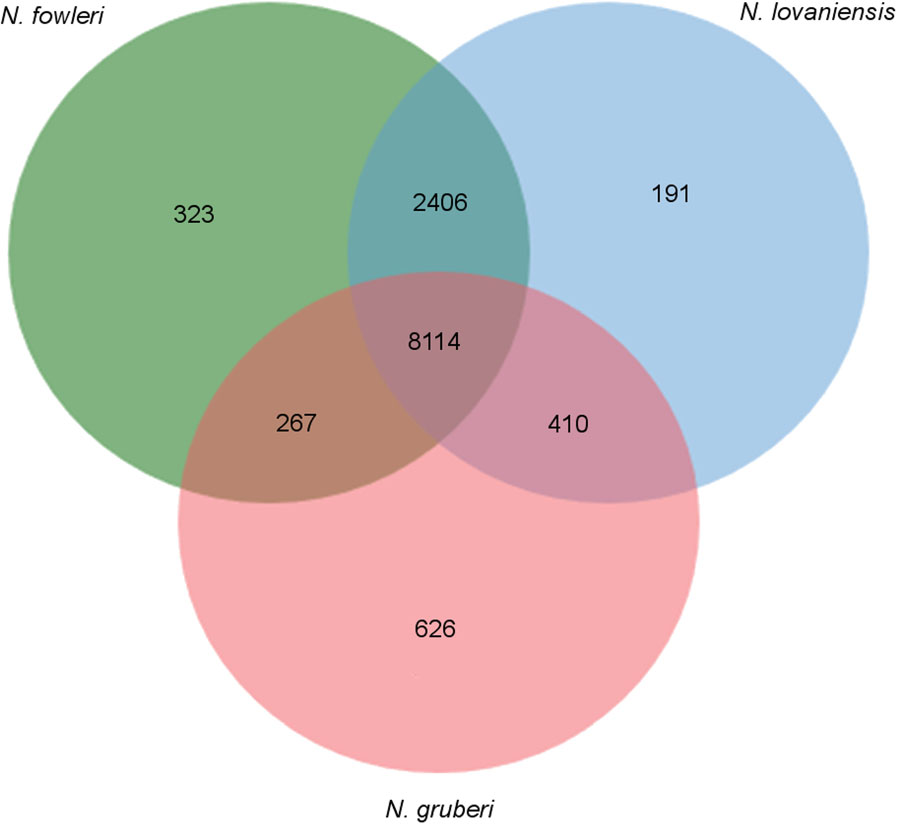
OrthoMCL Clustering of *Naegleria* proteins**.** To gain an overview of the diversity of the *Naegleria* protein repertoire, predicted protein of *N. lovaniensis* and *N. fowleri* as well as protein sequences of *N. gruberi* retrieved form Uniprot were clustered using OrthoMCL

To gain an overview of the protein diversity within the genus *Naegleria*, predicted proteins of all sequenced species were clustered into gene families based on BLAST similarities using OrthoMCL. 8114 gene families are shared among all *Naegleria* species and define the core genome. 2406 gene families are shared between *N. fowleri* and *N. lovaniensis*, while *N. fowleri* only shares 267 with *N. gruberi,* and *N. lovaniensis* shares 410 gene families with *N. gruberi*. Further, 191 gene families are specific for *N. lovaniensis*, 323 are specific for *N. fowleri* and 626 are specific for *N. gruberi* (Fig. 5).

To assess the function of protein clusters specific for *N. fowleri* in terms of pathogenicity, GO term enrichment analysis was performed using BiNGO. Regarding the category Biological Process, GO terms describing secretory processes and protein transport are enriched among *N. fowleri* specific proteins (Table 4). Further, the GO term membrane (GO:0016020, *p* value = 1.0188E-4) is significantly overrepresented in the category Cellular Component (Table 4). In contrast, analysing *N. lovaniensis* specific proteins, no significantly overrepresented term was found. In total, 97 proteins in the *N. fowleri* specific cluster are annotated with the GO term membrane. BLAST similarity search and PFAM Domain analysis of these proteins identified similarities to vesicular and cytoskeletal regulatory proteins including Rab and Rho small family GTPase related proteins as well as proteins containing an adenylate and guanylate cyclase catalytic domain (see Additional file 1).

**Table 4:** GO Enrichment of *N. fowleri* Specific Proteins (adj. *p*-value < 0.05) BiNGO

GO ID	Name	Adj. *P*-value
Biological Process		
GO:0009235	cobalamin metabolic process	4.1060E-2
GO:0050708	regulation of protein secretion	4.1060E-2
GO:0050709	negative regulation of protein secretion	4.1060E-2
GO:0051224	negative regulation of protein transport	4.1060E-2
Cellular Compartment		
GO:0016020	Membrane	1.0188E-4
Molecular Function		
GO:0004368	glycerol-3-phosphate dehydrogenase activity	3.7809E-2

## Discussion

Members of the genus *Naegleria* belong to a species-rich group of free-living eukaryotes. Besides the diversity within the genus, this protist can adapt to unfavorable environmental conditions by transforming to resting cysts or moving flagellates. As free-living eukaryotes with different life stages including facultative pathogenic species, *Naegleria* shows a high plasticity regarding morphology and pathogenicity. Regarding this feature, *Naegleria* is a powerful model to study fundamental eukaryotic processes including pathogenesis. Over decades, more than 40 *Naegleria* species were characterized based on ribosomal sequences, but large scale genomic approaches to unravel phylogenetic relationships are still missing [3, 48]. Here, we present the genome of *N. lovaniensis*, the closest relative of the human pathogenic amoeba *N. fowleri*. Assembling of PacBio data resulted in a nearly complete assembly of the *N. lovaniensis* genome. Based on the analysis of BUSCOs, the completeness of the assembly is comparable to the *N. fowleri* genome published in 2014. Furthermore, the assembly of *N. lovaniensis* is, with only 112 contigs, much less fragmented. Using the string graph assembler FALCON, we were able to fully assemble and circularize the mitochondrial sequence. Analysis of the mitochondrial gene repertoire shows high similarity with other annotated mitochondrial sequences within the genus of *Naegleria*. A typical *Naegleria* genome contains about 3000–5000 copies of an extrachromosomal rDNA plasmid [49]. Using long reads, the 15 kb rDNA plasmid could be reconstructed and rRNA genes were annotated. To summarize, the de novo sequenced genome of *N. lovaniensis* contains all characteristics of previous sequenced *Naegleria* species. By applying ab-initio gene prediction methods including RNAseq data, 15,195 proteins could be annotated and to 8749 (57.6%) at least one GO Term was mapped using Blast2GO annotation pipeline. A comparable number of predicted proteins is found in the *N. gruberi* genome. Proteins of *N. fowleri* were predicted based on transcriptome *de-novo* assembly using Trinity and TransDecoder to extract ORFs [8]. Trinity reports for each gene different isoforms, therefore predicted proteins based on TransDecoder may contain clusters of trinity isoforms. Further, TransDecoder is not considering start and stop codons. Therefore, the set of predicted proteins may contain incomplete proteins explaining the higher number reported for *N. fowleri*. With the description of the 30 Mb genome of *N. lovaniensis*, a further piece is added to reveal the complex taxonomic relationship of *Naegleria*. Using BLAST similarity genome networks and construction of a phylogenetic tree based on OrthoMCL single-copy orthologs, we confirm the close relationship of *N. lovaniensis* and *N. fowleri*. Within the genome similarity network, the *Naegleria* species share more genes with the human pathogenic amoeba *A. castelanii* than the closely related trypanosomes. Compared to free-living eukaryotes, obligate parasites including trypanosomes, show a functional reduction of their gene repertoire in adaption to the life cycle in the host, resulting for example in reduced metabolic capabilities [50]. The higher total number of *A. castelanii* proteins as well as functional protein similarities within free-living amoebas may explain the higher number of shared genes between *A. castelanii* and *Naegleria* in the genome similarity network. Based on the genomic data presented in this study and previous ribosomal sequence analysis, it is plausible that *N. fowleri* and *N. lovaniensis* evolved from a recent common ancestor [2, 51]. Due to the high similarity and the close relationship of the amoebas, knowledge of the *N. lovaniensis* genome provides the basis for further comparative studies to unravel pathogenicity factors of the human pathogenic *N. fowleri*. Transcriptomics. To gain insights in cellular pathways specific to *N. fowleri* and their involvement in pathogenesis, a closer look at *N. fowleri* specific proteins within the OrthoMCL clustering was taken. Characterizing the function of these proteins using GO enrichment analysis highlights the importance of the term membrane (GO:0016020) and the secretory protein system (GO:0050708, GO:0050709, GO:0051224). In concordance to our results, a comparative study of *N. fowleri* trophozoites with difference in pathogenicity levels shows an association of overexpressed proteins in highly pathogenic amoebas with the GO terms membrane and vesicles [8]. Already around 1990 *N. fowleri* has been compared to *N. lovaniensis* and other non-pathogenic *Naegleria* species to characterize pathogenicity factors. In contrast to weakly pathogenic *N. australiensis* and non-pathogenic *N. lovaniensis, N. fowleri* is more resistant to complement lysis when incubated with human serum [52]. Furthermore, only *N. fowleri,* but not *N. gruberi* shows membrane vesiculation during contact with human serum to eliminate MAC complex on its membrane [53]. A further comparison of *N. fowleri* with non-pathogenic Naegleria species and *Acanthamoeba species* identified a membrane protein (Mp2CL5), which is unique for *N. fowleri* and not expressed in other *Naegleria* species. It is localized at pseudopod structures and likely involved in attachment or sensing of nutrient and other external environmental influences [54]. Comparing the adhesion to extracellular matrix structures of pathogenic and non-pathogenic *Naegleria* shows a strong attachment of *N. fowleri* while a weaker interaction is reported for *N. lovaniensis*. In addition, the formation of focal adhesion structures containing actin and integrin-like proteins is observed in *N. fowleri* but not for *N. lovaniensis.* Furthermore, a 70 kDa protein species was found to be expressed at higher levels ins *N. fowleri* [55]. Attachment to the host cell is a crucial point in the pathogenesis of *N. fowleri*. In this context, an integrin-like membrane protein binding to fibronectin was identified and the formation of actin structures regulated by protein kinase C in response to fibronectin was shown in *N. fowleri* trophozoites [56]. Altogether, expression of membrane proteins and regulation of membrane structures either involved in attachment to the host membrane or defending against lysis, are important mechanisms of pathogenesis. Vesicular transport and regulation of protein secretion are important points in the pathogenesis of *N. fowleri* and is supported by the GO enrichment analysis. Beside the above mentioned membrane vesiculation, recent studies identified secreted cysteine and metalloproteinases which are involved in the degradation of the host extracellular matrix and enable the penetration of the blood-brain barrier [57–59].

Among others, BLAST similarity search of *N. fowleri* specific proteins associated with the GO term membrane identified different Rab and Rho family small GTPase proteins. Furthermore, a recent proteomics study identified an upregulation of the Rho guanine nucleotide exchange factor 28, which is involved in actin regulation, in highly pathogenic *N. fowleri* [60]. Cytoskeleton changes and the formation of actin-rich structures in response to extracellular matrix protein has also been reported for *E. histolytica.* There, adhesion to fibronectin may induce formation of further attachment structures and the secretion of proteolytic enzymes [61]*.* A recent study in *E. histolytica* shows the role of Rab21-mediated actin cytoskeleton changes and the involvement of the Rab GTPase in the formation of actin dots during invasion of the extracellular matrix [62]. Rab GTPases are not only known as the master regulators in vesicular transport but are also involved in the regulation of actin cytoskeleton changes. Regarding the pathogenicity of *N. fowleri*, Rab GTPases might not only play an important role in the regulation of vesicular transport and secretion of proteases but also in the formation of actin structures promoting adhesion to the host cell.

## Conclusion

Sequencing and de novo assembly of the *N. lovaniensis* genome supports the hypothesis of the close relationship to the human pathogenic amoeba *N. fowleri.* Thus, knowledge of the *N. lovaniensis* genome provides the basis for a more detailed interspecies comparison on a genomic, proteomic or molecular level to unravel pathways involved in the pathogenicity of PAM and to identify potential structures for possible treatment options.

## Additional files

Additional file 1: *N. fowleri* specific Proteins associated with the GO Term “Membrane”. Results of PFAM domain analysis and BLAST search against SwissProt of *N. fowleri* specific proteins associated with the GO Term “Membrane”. (XLSX 17 kb)

Additional file 2: Genome Similarity Network: Number of shared gene families between unicellular eukaryotic species within the Genome Similarity Network. (XLSX 9 kb)

## Abbreviations

BUSCO: Benchmarking Universal Single-Copy Ortholog
EGN: Evolutionary Gene and Genome Network
GO: Gene Ontology
LTRs: Long-terminal Repeat
NGS: Next Generation Sequencing
ORF: Open Reading frame
PAM: Primary amoebic meningoencephalitis
PBS: Phosphate-buffered saline
RNAseq: RNA sequencing

## References

[1] Schuster FL, Visvesvara GS (2004). Free-living amoebae as opportunistic and non-opportunistic pathogens of humans and animals. Int J Parasitol.

[2] De Jonckheere JF (2014). What do we know by now about the genus Naegleria?. Exp Parasitol.

[3] De Jonckheere JF (2004). Molecular definition and the ubiquity of species in the genus Naegleria. Protist.

[4] Marciano-Cabral F, Cabral G a (2007). The immune response to Naegleria fowleri amebae and pathogenesis of infection. FEMS Immunol Med Microbiol.

[5] Khan NA (2012). Is ritual cleansing a missing link between fatal infection. Clin Infectous Dis.

[6] Visvesvara GS, Moura H, Schuster FL (2007). Pathogenic and opportunistic free-living amoebae: Acanthamoeba spp., Balamuthia mandrillaris, Naegleria fowleri, and Sappinia diploidea. FEMS Immunol Med Microbiol.

[7] Sriram R, Noman F, Ali F (2011). Primary amebic meningoencephalitis caused by Naegleria fowleri, Karachi, Pakistan. Emerg Infect Dis.

[8] Zysset-Burri DC, Müller N, Beuret C, Heller M, Schürch N, Gottstein B (2014). Genome-wide identification of pathogenicity factors of the free-living amoeba Naegleria fowleri. BMC Genomics.

[9] Fritz-Laylin LK, Prochnik SE, Ginger ML, Dacks JB, Carpenter ML, Field MC (2010). The genome of Naegleria gruberi illuminates early eukaryotic versatility. Cell.

[10] Cervantes-Sandoval I, Serrano-Luna JJ, Pacheco-Yépez J, Silva-Olivares A, Tsutsumi V, Shibayama M (2010). Differences between Naegleria fowleri and Naegleria gruberi in expression of mannose and fucose glycoconjugates. Parasitol Res.

[11] Serrano-Luna J, Cervantes-Sandoval I, Tsutsumi V, Shibayama M (2007). A biochemical comparison of proteases from pathogenic Naegleria fowleri and non-pathogenic Naegleria gruberi. J Eukaryot Microbiol.

[12] Herman EK, Greninger AL, Visvesvara GS, Marciano-Cabral F, Dacks JB, Chiu CY (2013). The Mitochondrial Genome and a 60-kb Nuclear DNA Segment from *Naegleria fowleri* , the Causative Agent of Primary Amoebic Meningoencephalitis. J Eukaryot Microbiol.

[13] Koren S, Walenz BP, Berlin K, Miller JR, Bergman NH, Phillippy AM (2017). Canu: scalable and accurate long-read assembly via adaptive k-mer weighting and repeat separation. Genome Res.

[14] Chin C-S, Peluso P, Sedlazeck FJ, Nattestad M, Concepcion GT, Clum A (2016). Phased diploid genome assembly with single-molecule real-time sequencing. Nat Methods.

[15] Burri DC, Gottstein B, Zumkehr B, Hemphill A, Schürch N, Wittwer M (2012). Development of a high- versus low-pathogenicity model of the free-living amoeba Naegleria fowleri. Microbiology.

[16] Myers G. Efficient Local Alignment Discovery amongst Noisy Long Reads. In: Brown D, Morgenstern B, editors. Algorithms in Bioinformatics. WABI 2014. Springer, Berlin, Heidelberg. Lect. Notes Comput. Sci. 2014;8701:52–67.

[17] Chin C-S, Alexander DH, Marks P, Klammer AA, Drake J, Heiner C (2013). Nonhybrid, finished microbial genome assemblies from long-read SMRT sequencing data. Nat Methods.

[18] Simão FA, Waterhouse RM, Ioannidis P, Kriventseva EV, Zdobnov EM (2015). BUSCO: assessing genome assembly and annotation completeness with single-copy orthologs. Bioinformatics.

[19] Simão FA, Waterhouse RM, Ioannidis P, Kriventseva EV, Zdobnov EM (2016). Eukaryota Dataset ODB9.

[20] Li, H. Minimap2: pairwise alignment for nucleotide sequences. Bioinformatics. 2018; 10.1093/bioinformatics/bty191 10.1093/bioinformatics/bty191 PMC6137996 29750242

[21] Nawrocki EP, Kolbe DL, Eddy SR (2009). Infernal 1.0: inference of RNA alignments. Bioinformatics.

[22] Smit AFA, Hubley R (2015). RepeatModeler Open-1.0. RepeatModeler Open-1.0.8.

[23] Bao Z, Eddy SR (2002). Automated de novo identification of repeat sequence families in sequenced genomes. Genome Res.

[24] Price AL, Jones NC, Pevzner PA (2005). De novo identification of repeat families in large genomes. Bioinformatics.

[25] Eddy SR (2011). Accelerated Profile HMM Searches. PLoS Comput Biol.

[26] Abrusán G, Grundmann N, DeMester L, Makalowski W (2009). TEclass--a tool for automated classification of unknown eukaryotic transposable elements. Bioinformatics.

[27] Smit A, Hubley R, Green P. RepeatMasker Open-4.0. 2013-2015. http://www.repeatmasker.org.

[28] Hoff KJ, Lange S, Lomsadze A, Borodovsky M, Stanke M (2016). BRAKER1: unsupervised RNA-Seq-based genome annotation with GeneMark-ET and AUGUSTUS: table 1. Bioinformatics.

[29] Lomsadze A, Burns PD, Borodovsky M (2014). Integration of mapped RNA-Seq reads into automatic training of eukaryotic gene finding algorithm. Nucleic Acids Res.

[30] Stanke M, Diekhans M, Baertsch R, Haussler D (2008). Using native and syntenically mapped cDNA alignments to improve de novo gene finding. Bioinformatics.

[31] Griffiths-Jones S (2003). Rfam: an RNA family database. Nucleic Acids Res.

[32] Bateman A, Martin MJ, O’Donovan C, Magrane M, Alpi E, Antunes R (2017). UniProt: the universal protein knowledgebase. Nucleic Acids Res.

[33] Conesa A, Götz S, García-Gómez JM, Terol J, Talón M, Robles M (2005). Blast2GO: a universal tool for annotation, visualization and analysis in functional genomics research. Bioinformatics.

[34] Finn RD, Coggill P, Eberhardt RY, Eddy SR, Mistry J, Mitchell AL (2016). The Pfam protein families database: towards a more sustainable future. Nucleic Acids Res.

[35] Halary S, McInerney JO, Lopez P, Bapteste E (2013). EGN: a wizard for construction of gene and genome similarity networks. BMC Evol Biol.

[36] Shannon P (2003). Cytoscape: a software environment for integrated models of biomolecular interaction networks. Genome Res.

[37] Li L, Stoeckert CJ, Roos DS (2003). OrthoMCL: identification of Ortholog groups for eukaryotic genomes. Genome Res.

[38] Edgar RC (2004). MUSCLE: multiple sequence alignment with high accuracy and high throughput. Nucleic Acids Res.

[39] Capella-Gutiérrez S, Silla-Martínez JM, Gabaldón T (2009). trimAl: A tool for automated alignment trimming in large-scale phylogenetic analyses. Bioinformatics.

[40] Kück P, Meusemann K (2010). FASconCAT: convenient handling of data matrices. Mol Phylogenet Evol.

[41] Darriba D, Taboada GL, Doallo R, Posada D. ProtTest-HPC: Fast Selection of Best-Fit Models of Protein Evolution. In: Guarracino MR. et al., editors. Euro-Par 2010 Parallel Processing Workshops. Euro-Par 2010. Springer, Berlin, Heidelberg. Lect. Notes Comput. Sci. 2011;6586:177–84.

[42] Stamatakis A (2014). RAxML version 8: a tool for phylogenetic analysis and post-analysis of large phylogenies. Bioinformatics.

[43] Rambaut A (2016). FigTree v1. 4.0. A graphical viewer of phylogenetic trees.

[44] Maere S, Heymans K, Kuiper M (2005). BiNGO: a Cytoscape plugin to assess overrepresentation of gene ontology categories in biological networks. Bioinformatics.

[45] Zdobnov EM, Tegenfeldt F, Kuznetsov D, Waterhouse RM, Simão FA, Ioannidis P (2017). OrthoDB v9.1: cataloging evolutionary and functional annotations for animal, fungal, plant, archaeal, bacterial and viral orthologs. Nucleic Acids Res.

[46] Fritz-Laylin LK, Ginger ML, Walsh C, Dawson SC, Fulton C (2011). The Naegleria genome: a free-living microbial eukaryote lends unique insights into core eukaryotic cell biology. Res Microbiol.

[47] Clark CG, Cross GAM, De Jonckheere JF (1989). Evaluation of evolutionary divergence in the genus Naegleria by analysis of ribosomal DNA restriction enzyme patterns. Mol Biochem Parasitol.

[48] De Jonckheere JF (2002). A century of research on the Amoeboflagellate genus Naegleria. Acta Protozool.

[49] Clark CG, Cross GA (1987). rRNA genes of Naegleria gruberi are carried exclusively on a 14-kilobase-pair plasmid. Mol Cell Biol.

[50] Jackson AP (2015). Genome evolution in trypanosomatid parasites. Parasitology.

[51] De Jonckheere JF (2011). Origin and evolution of the worldwide distributed pathogenic amoeboflagellate Naegleria fowleri. Infect Genet Evol.

[52] Whiteman LY, Marciano-Cabral F (1987). Susceptibility of pathogenic and nonpathogenic Naegleria spp to complement-mediated lysis. Infect Immun.

[53] Toney DM, Marciano-Cabral F (1994). Membrane vesiculation of Naegleria fowleri amoebae as a mechanism for resisting complement damage. J Immunol.

[54] Réveiller FL, Suh SJ, Sullivan K, Cabanes PA, Marciano-Cabral F (2001). Isolation of a unique membrane protein from Naegleria fowleri. J Eukaryot Microbiol.

[55] Jamerson M, da Rocha-Azevedo B, Cabral GA, Marciano-Cabral F (2012). Pathogenic Naegleria fowleri and non-pathogenic Naegleria lovaniensis exhibit differential adhesion to, and invasion of, extracellular matrix proteins. Microbiology.

[56] Han KL, Lee HJ, Myeong HS, Shin HJ, Im KI, Park SJ (2004). The involvement of an integrin-like protein and protein kinase C in amoebic adhesion to fibronectin and amoebic cytotoxicity. Parasitol Res.

[57] Vyas IK, Jamerson M, Cabral G a, Marciano-Cabral F (2015). Identification of peptidases in highly pathogenic vs. weakly pathogenic Naegleria fowleri Amebae. J Eukaryot Microbiol.

[58] Lam C, Jamerson M, Cabral G, Carlesso AM, Marciano-Cabral F (2017). Expression of matrix metalloproteinases in Naegleria fowleri and their role in invasion of the central nervous system. Microbiology.

[59] Coronado-Velázquez D, Betanzos A, Serrano-Luna J, Shibayama M. An in vitro model of the blood-brain barrier: Naegleria fowleri affects the tight junction proteins and activates the microvascular endothelial cells. J Eukaryot Microbiol. 2018;(0):1–16. 10.1111/jeu.12522 29655298

[60] Jamerson M, Schmoyer JA, Park J, Marciano-Cabral F, Cabral GA (2017). Identification of Naegleria fowleri proteins linked to primary amoebic meningoencephalitis. Microbiology.

[61] Meza I (2000). Extracellular matrix-induced signaling in entamoeba histolytica: its role in invasiveness. Parasitol Today.

[62] Emmanuel M, Nakano YS, Nozaki T, Datta S (2015). Small GTPase Rab21 mediates fibronectin induced actin reorganization in Entamoeba histolytica: implications in pathogen invasion. PLoS Pathog.

